# Supplementary material for: Impaired stem cell migration and divisions in Duchenne muscular dystrophy revealed by live imaging
Source: Nat Commun. 2026 Jan 28;17:1769. doi: 10.1038/s41467-026-68474-5 (PMC12917047; doi:10.1038/s41467-026-68474-5)
Supplement: Supplementary file 1 — Supplementary Information [file 41467_2026_68474_MOESM1_ESM.pdf]

## Supplementary Information for:

### Impaired stem cell migration and divisions in Duchenne Muscular Dystrophy revealed by live imaging

Liza Sarde, Gaëlle Letort, Hugo Varet, Vincent Laville, Julien Fernandes, Shahragim Tajbakhsh, Brendan Evano

1. Supplementary Figures 1-8
2. Supplementary Tables 1-2

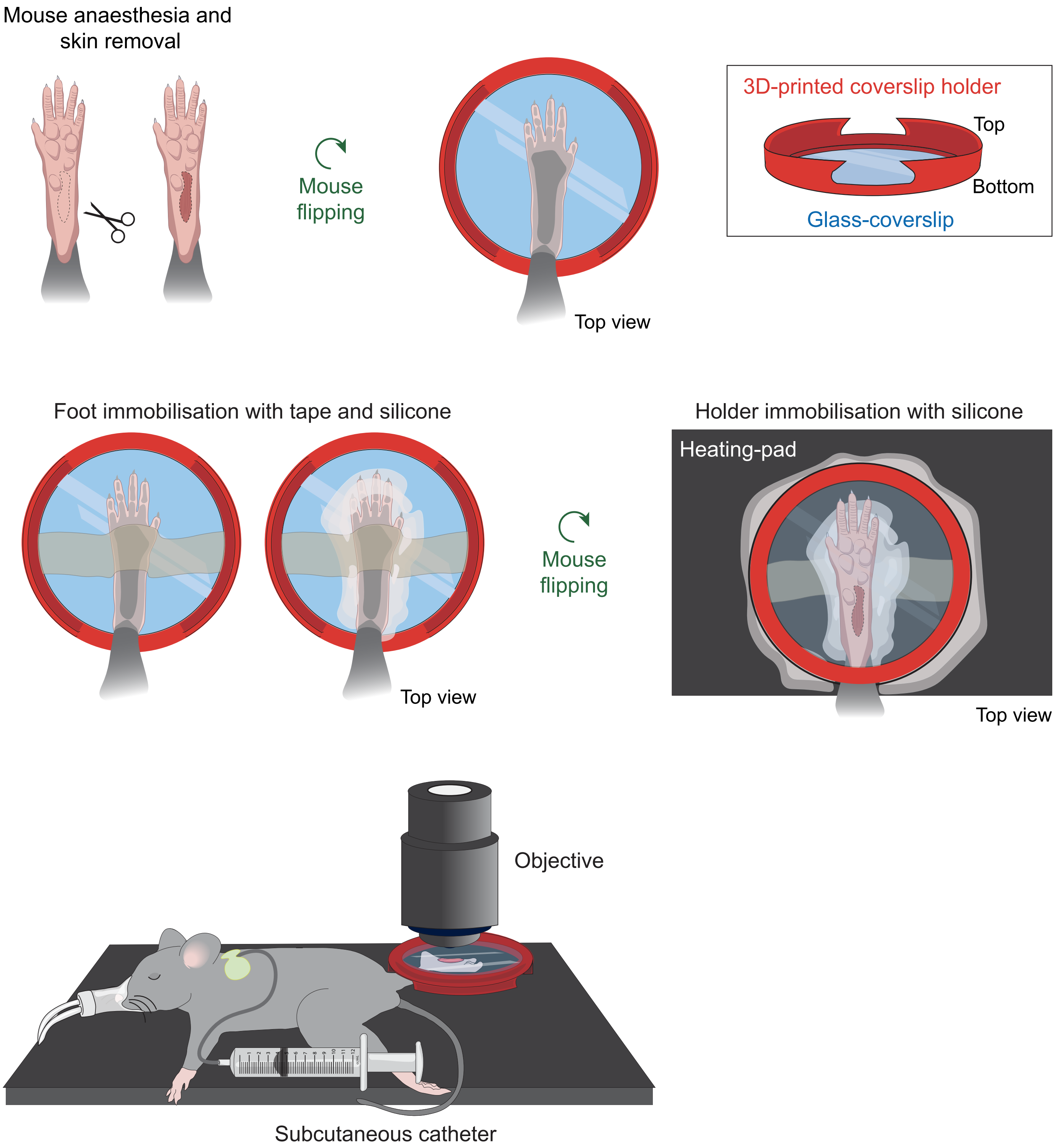


**Supplementary Figure 1. Intravital imaging setup.** Related to Figure 1.

The mouse was anaesthetised with isofluorane, kept on a heat pad and hydrated every hour with NaCl 0.9% *via* a catheter. A small piece of the mouse skin on foot was removed to expose the FDB. The foot was immobilised with tape and silicone to a glass-coverslip attached to a 3D-printed custom coverslip holder. The holder was fixed to a heating-pad (silicone) and the

mouse was hydrated with 0.9% NaCl *via* a catheter, enabling continuous intravital imaging up to 10h. See also Methods.

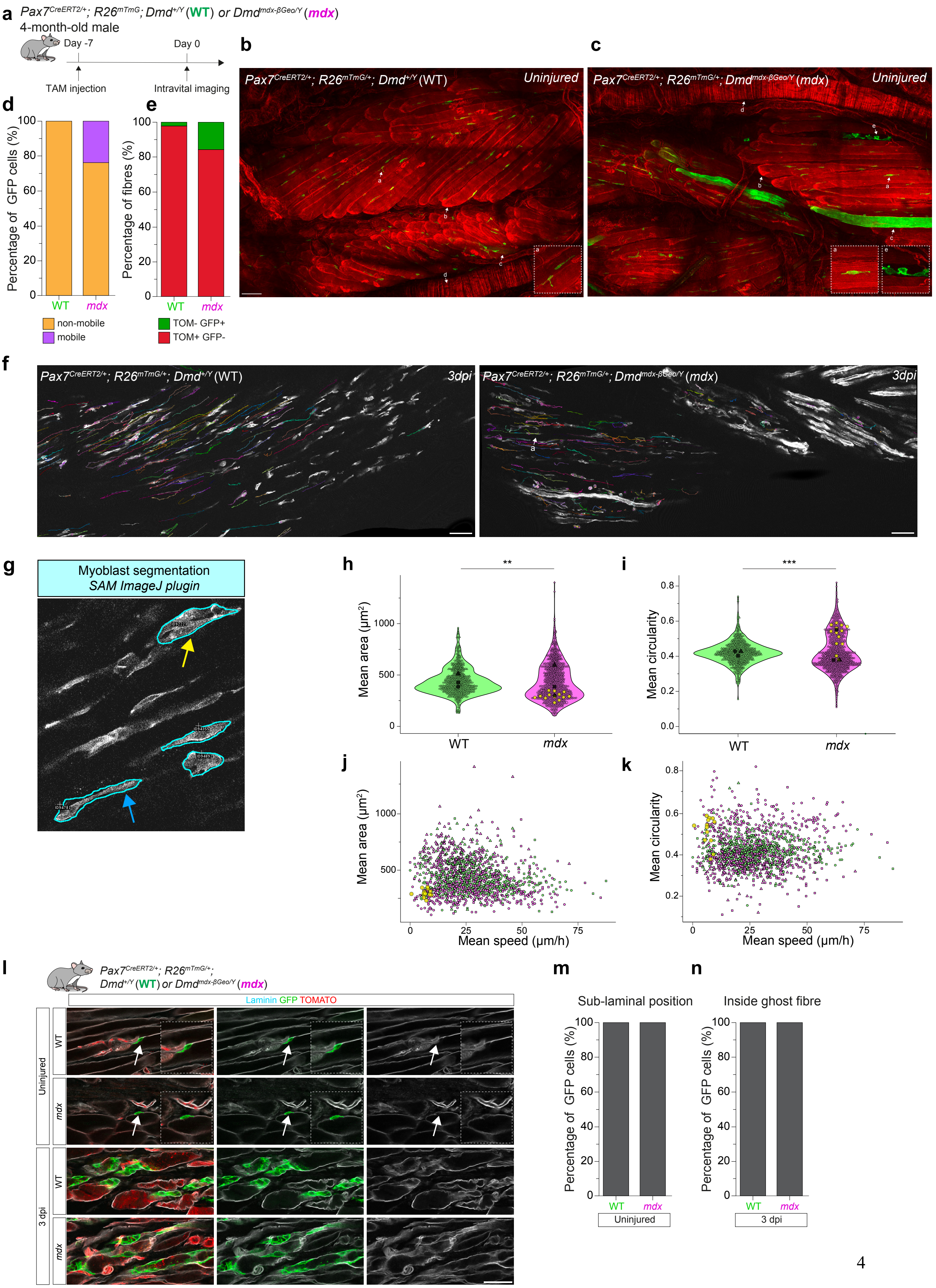

a Pax7CreERT2/+; R26mTmG; Dmd+/Y (WT) or Dmdmdx-βGeo/Y (mdx)
4-month-old male
Day -7
Day 0
TAM injection
Intravital imaging
b Pax7CreERT2/+; R26mTmG/+; Dmd+/Y (WT) Uninjured
c Pax7CreERT2/+; R26mTmG/+; Dmdmdx-βGeo/Y (mdx) Uninjured
d Percentage of GFP cells (%)
WT mdx
non-mobile
mobile
e Percentage of fibres (%)
TOM- GFP+
TOM+ GFP-
f Pax7CreERT2/+; R26mTmG/+; Dmd+/Y (WT) 3dpi
Pax7CreERT2/+; R26mTmG/+; Dmdmdx-βGeo/Y (mdx) 3dpi
g Myoblast segmentation
SAM ImageJ plugin
h Mean area (µm²) **
i Mean circularity ***
j Mean area (µm²)
Mean speed (µm/h)
k Mean circularity
Mean speed (µm/h)
l Pax7CreERT2/+; R26mTmG/+;
Dmd+/Y (WT) or Dmdmdx-βGeo/Y (mdx)
Laminin GFP TOMATO
Uninjured
3 dpi
WT
mdx
m Sub-laminal position
Percentage of GFP cells (%)
Uninjured
n Inside ghost fibre
Percentage of GFP cells (%)
3 dpi

**Supplementary Figure 2.** Related to Figure 1.

**(a)** Experimental scheme. Also see Methods.

**(b-c)** Representative example of intravital imaging of uninjured FDB muscles of WT (**b**) and *mdx* (**c**) mice. Arrows: a, MuSC with 1 or several projections; b, intact FDB fibre (GFP-); c, regenerated fibre (GFP+); d, collagen rich region; e, activated myoblast. Scale bar, 50 µm. See Movie S1 and S2.

**(d)** Quantification of mobile *vs* non-mobile MuSCs from WT and *mdx* mice in uninjured muscles. N = 1 experiment. n = 83 WT cells, n = 83 *mdx* cells.

**(e)** Quantification of GFP+ vs GFP- muscle fibres from WT and *mdx* mice in uninjured muscles. N = 1 experiment. n = 98 WT fibres, n = 63 *mdx* fibres.

**(f)** Cell tracking overlays of FDB muscles at 3 dpi from WT (left) and *mdx* (right) mice visualised by intravital imaging. Scale bar, 50 µm. See Movies S5 and S6.

**(g)** Representative image of MuSC segmentation with ImageJ SAM plugin[32] from FDB muscles at 3 dpi visualised by intravital imaging. Segmentation was performed on tracked cells. Segmented objects are displayed in cyan. Arrows indicate segmented objects with a single (blue arrow) or several (yellow arrow) cells.

**(h)** Mean area of SAM-segmented WT and *mdx* MuSCs.

**(i)** Mean circularity of SAM-segmented WT and *mdx* MuSCs.

**(j)** Relationship between mean area and mean migration speed of SAM-segmented WT and *mdx* MuSCs.

**(k)** Relationship between mean circularity and mean migration speed of SAM-segmented WT and *mdx* MuSCs.

h-k: Yellow dots indicate *mdx* cells visually identified as small, round and immobile (Fig. 1d and Movie S4) as reference.

**(l)** Laminin immunostaining of FDB muscles (longitudinal sections), uninjured or at 3 dpi, of WT and *mdx* 4 mice. Arrows indicate MuSCs in sub-laminal position. Scale bar, 50 µm.

**(m)** Quantification of MuSCs in sub-laminal position in uninjured FDB muscles of WT and *mdx* mice. N = 1 experiment. n = 30 WT cells, n = 30 *mdx* cells.

**(n)** Quantification of MuSCs located within ghost (injured) fibres and below the basal lamina in injured FDB muscles (3 dpi) WT and *mdx* mice. N = 1 experiment. n = 50 WT cells, n = 50 *mdx* cells.

Statistical tests: **(h,i)** Two-sided test from linear mixed models. Data are presented as mean values (large symbols) and individual values (small symbols). * $p < 0.05$, ** $p < 0.01$, *** $p < 0.001$. Source data are provided as a Source Data file.

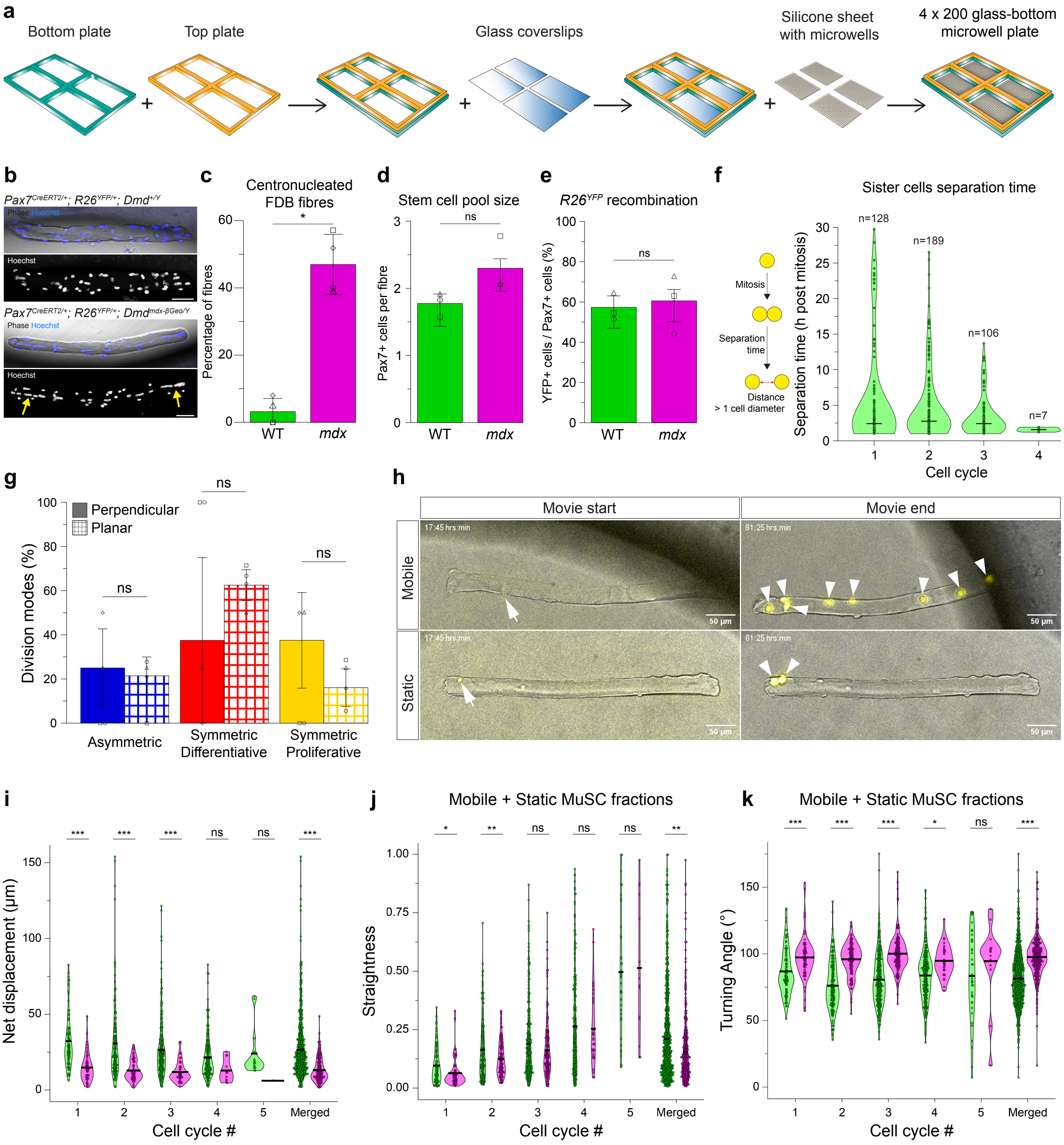


**Supplementary Figure 3.** Related to Figure 2.

**(a)** Microwell plate assembly. Also see Methods.

**(b)** Immunostaining of FDB fibres from WT and *mdx* mice. Arrows indicate myonuclei in central position. Scale bar, 50 µm.

**(c)** Percentage of centronucleated FDB fibres from WT and *mdx* mice. N = 4 experiments. p = 0.029.

**(d)** Number of PAX7 positive MuSCs per FDB fibre from WT and *mdx* mice. Analysis performed immediately upon isolation. N = 3 experiments. p = 0.055.

**(e)** Recombination efficiency of $Pax7^{CreERT2/+}$; $R26^{YFP/+}$ from FDB fibres of WT and *mdx* mice. Analysis performed immediately upon isolation. N = 3 experiments. p = 0.88.

**(f)** Timing of sister cell separation (cell-cell distance > 1 cell diameter) after mitosis. N = 4 experiments, n = number of analysed divisions.

**(g)** Modes of cell divisions of YFP-labelled myogenic *mdx* cells following perpendicular or planar divisions. N = 4 experiments (represented by different geometrical shapes). p-values: ACD (planar vs perpendicular) = 0.82; SCDd (planar vs perpendicular) = 0.19; SCDp (planar vs perpendicular) = 0.16. Data are presented as mean values +/- SD.

**(h)** Representative example of mobile (top) and static (bottom) migration of YFP-labelled myogenic cells (see Movie S8).

**(i)** Net distance of mobile fraction of WT and *mdx* YFP-labelled myogenic cells, for individual and merged cell cycles. N = 4 experiments. p(CellCycle1) = 2.17e-02; p(CellCycle2) = 6.23e-01; p(CellCycle3) = 7.41e-01; p(CellCycle4) = 3.77e-01, p(CellCycle5) = 3.71e-02; p(Merged) = 0.191.

**(j)** Straightness of mobile and static fractions of WT and *mdx* YFP-labelled myogenic cells, for individual and merged cell cycles. N = 4 experiments.

**(k)** Turning angle of mobile and static fractions of WT and *mdx* YFP-labelled myogenic cells, for individual and merged cell cycles. N = 4 experiments.

Statistical tests: **(c-e)** Two-sided Wilcoxon test, data are presented as mean values +/- SD; **(g, i-k)** Two-sided test from Linear mixed models with **(i-k)** Tukey's correction. Horizontal lines in violin plots represent the mean. * $p < 0.05$, ** $p < 0.01$, *** $p < 0.001$. Source data are provided as a Source Data file.

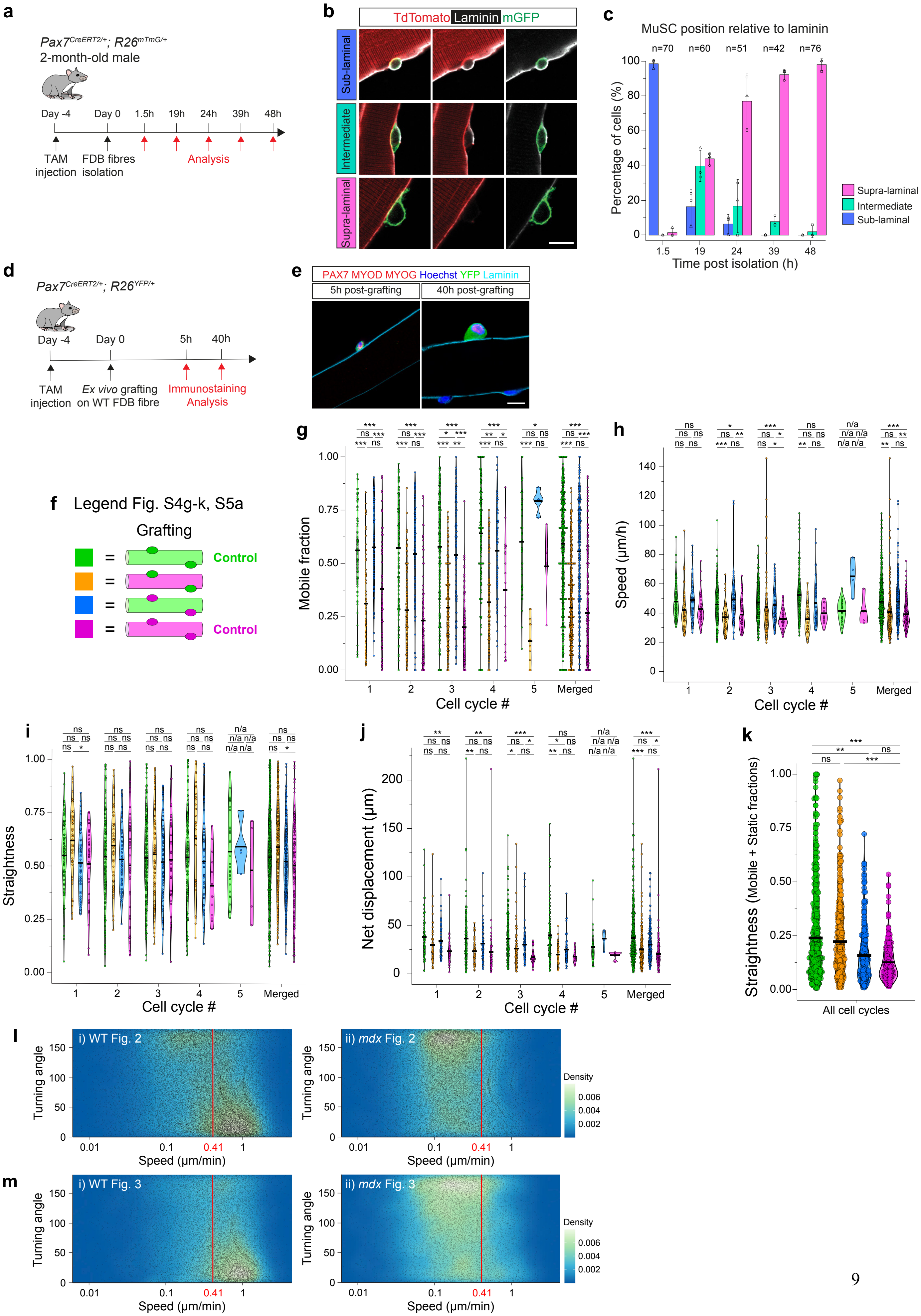
a
Pax7CreERT2/+; R26mTmG/+
2-month-old male
Day -4
Day 0
1.5h
19h
24h
39h
48h
TAM injection
FDB fibres isolation
Analysis
b
TdTomato Laminin mGFP
Sub-laminal
Intermediate
Supra-laminal
c
MuSC position relative to laminin
n=70 n=60 n=51 n=42 n=76
Percentage of cells (%)
Time post isolation (h)
Supra-laminal
Intermediate
Sub-laminal
d
Pax7CreERT2/+; R26YFP/+
Day -4
Day 0
5h
40h
TAM injection
Ex vivo grafting on WT FDB fibre
Immunostaining Analysis
e
PAX7 MYOD MYOG Hoechst YFP Laminin
5h post-grafting
40h post-grafting
f
Legend Fig. S4g-k, S5a
Grafting
Control
Control
g
Mobile fraction
Cell cycle #
Merged
h
Speed (µm/h)
Cell cycle #
Merged
i
Straightness
Cell cycle #
Merged
j
Net displacement (µm)
Cell cycle #
Merged
k
Straightness (Mobile + Static fractions)
All cell cycles
l
i) WT Fig. 2
ii) mdx Fig. 2
Turning angle
Speed (µm/min)
Density
m
i) WT Fig. 3
ii) mdx Fig. 3
Turning angle
Speed (µm/min)
Density

**Supplementary Figure 4.** Related to Figure 3.

**(a)** Experimental scheme. *Pax7*$^{CreERT2/+}$; *R26*$^{mTmG/+}$ WT 2-month-old male mice were treated with tamoxifen to label MuSCs with GFP. FDB fibres were isolated, cultured, fixed and stained for Laminin (basal lamina) at indicated time points post-isolation.

**(b)** Representative example of sub-/supra-laminal or intermediate MuSC positions relative to basal lamina.

**(c)** Percentage of sub-/supra-laminal or intermediate MuSC positions relative to basal lamina at different timepoints post-isolation. N = 3 experiment, n = analysed number of MuSCs. Data are presented as mean values +/- SD.

**(d)** Experimental scheme. *Pax7*$^{CreERT2/+}$; *R26*$^{YFP/+}$ 2-month-old male mice were treated with tamoxifen to label MuSCs with YFP. YFP+ MuSCs were grafted *ex vivo* on recipient FDB fibres, cultured, fixed and stained for Laminin (basal lamina), Pax7/Myod/Myog and YFP at indicated time points post-grafting.

**(e)** Representative examples of Laminin, Pax7/Myod/Myog and YFP immunostaining of *ex vivo* grafted MuSCs at 5h and 40h post-grafting. Scale bar = 10 µm.

**(f)** Colour code of 4 grafting conditions (WT Ctrl, WT_to_*mdx, mdx*_to_WT, *mdx* Ctrl).

**(g)** Mobile fraction for all grafting conditions for individual and merged cell cycles. N = 5 experiments.

**(h)** Migration speed of mobile fraction for all grafting conditions for individual and merged cell cycles. N = 5 experiments.

**(i)** Migration straightness of mobile fraction for all grafting conditions for individual and merged cell cycles. N = 5 experiments.

**(j)** Net displacement of mobile fraction for all grafting conditions, for individual and merged cell cycles. N = 5 experiments.

**(k)** Migration straightness of mobile and static fractions for all grafting conditions, for merged cell cycles. N = 5 experiments.

**(l)** Threshold value between mobile and static fractions (Fig. 2 dataset). Density plots of turning angle over instantaneous speed for endogenous i) WT and ii) *mdx* MuSCs.

**(m)** Threshold value between mobile and static fractions (Fig. 3 dataset). Density plots of turning angle over instantaneous speed for control grafting conditions i) WT Ctrl and ii) *mdx* Ctrl.

Statistical tests: **(g-k)** Two-sided test from Linear mixed models with Tukey's correction. Horizontal lines in violin plot represent mean. * $p < 0.05$, ** $p < 0.01$, *** $p < 0.001$. Source data are provided as a Source Data file.

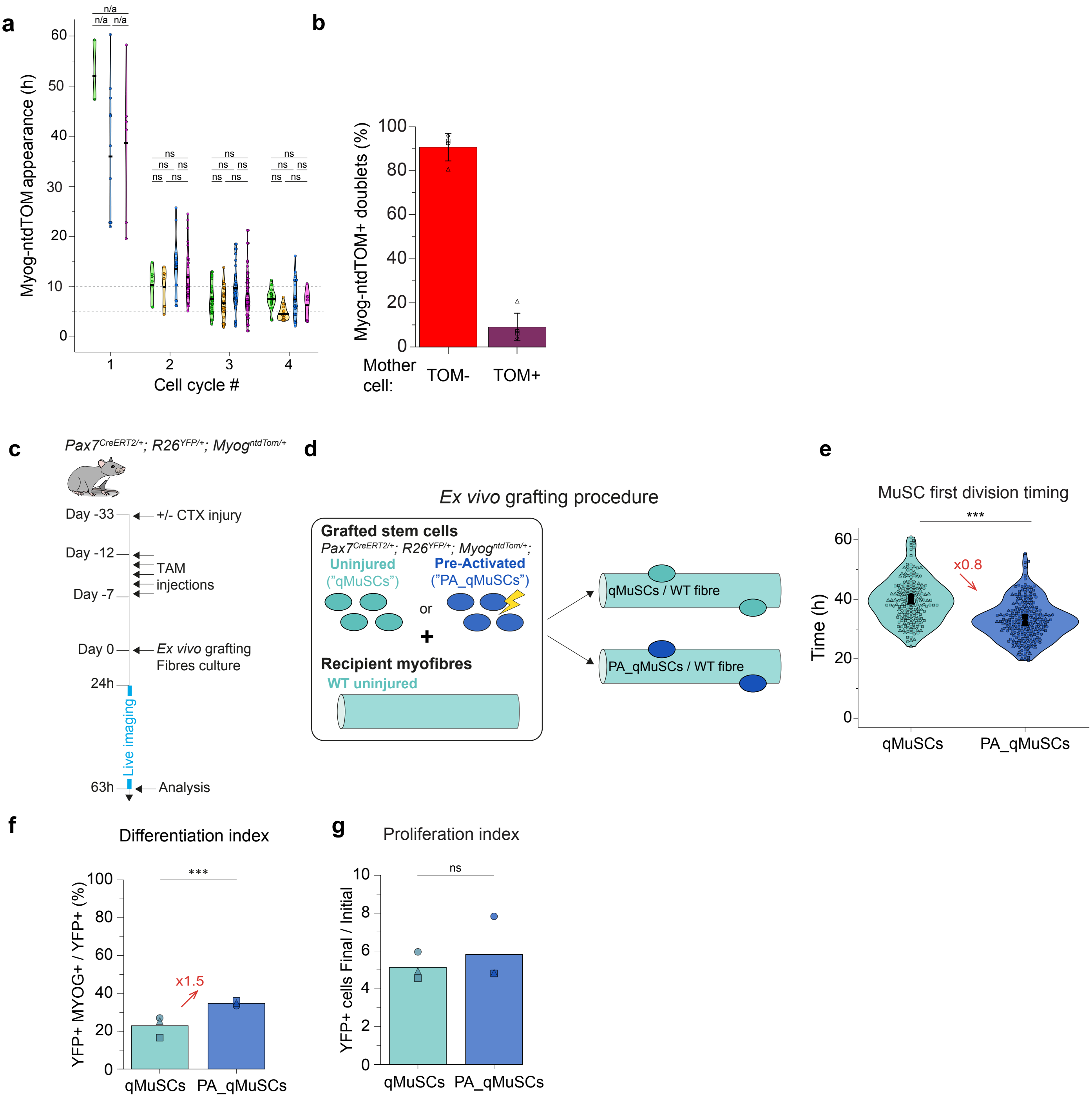


**Supplementary Figure 5.** Related to Figure 3.

**(a)** Timing of *Myog*$^{ntdTOM}$ reporter appearance for each cell cycle, for all grafting conditions. N = 5 experiments.

**(b)** Percentage of SCDd generating TOM+ doublet coming from a Myog-ntdTOM-positive or -negative mother cell. N = 5 experiments. Data are presented as mean values +/- SD.

**(c)** Experimental scheme. FDB muscles of *Pax7*$^{CreERT2/+}$*; R26*$^{YFP/+}$*; Myog*$^{ntdTom/+}$ mice were left intact or injured with cardiotoxin. MuSCs were YFP-labelled between 21 to 28 days post injury, grafted *ex vivo* on recipient WT FDB fibres, cultured and filmed for 63h.

**(d)** *Ex vivo* grafting procedure of MuSCs from uninjured (qMuSCs) or regenerated (Pre-Activated, PA_qMuSCs) FDB muscles to recipient WT FDB fibres.

**(e)** First division timing of qMuSCs and PA_qMuSCs upon e*x vivo* grafting to FDB fibres. n = 180 qMuSCs, n = 216 PA_qMuSCs. N = 3 experiments (represented by different geometrical shapes). p = 4.27e-215.

**(f)** Differentiation index of qMuSCs and PA_qMuSCs upon e*x vivo* grafting to FDB fibres at 63h post-grafting. n = 131 qMuSCs, n = 135 PA_qMuSCs. N = 3 experiments (represented by different geometrical shapes). p = 7.07e-14.

**(g)** Proliferation index of qMuSCs and PA_qMuSCs upon e*x vivo* grafting to FDB fibres at 63h post-grafting. n = 136 qMuSCs, n = 134 PA_qMuSCs. N = 3 experiments (represented by different geometrical shapes). p = 9.70e-12.

Statistical tests: **(a,e-g)** Two-sided test from Linear mixed models. Data are presented as mean values large symbols) and individual values (small symbols). Horizontal lines in violin plot represent mean. * $p < 0.05$, ** $p < 0.01$, *** $p < 0.001$. Source data are provided as a Source Data file.

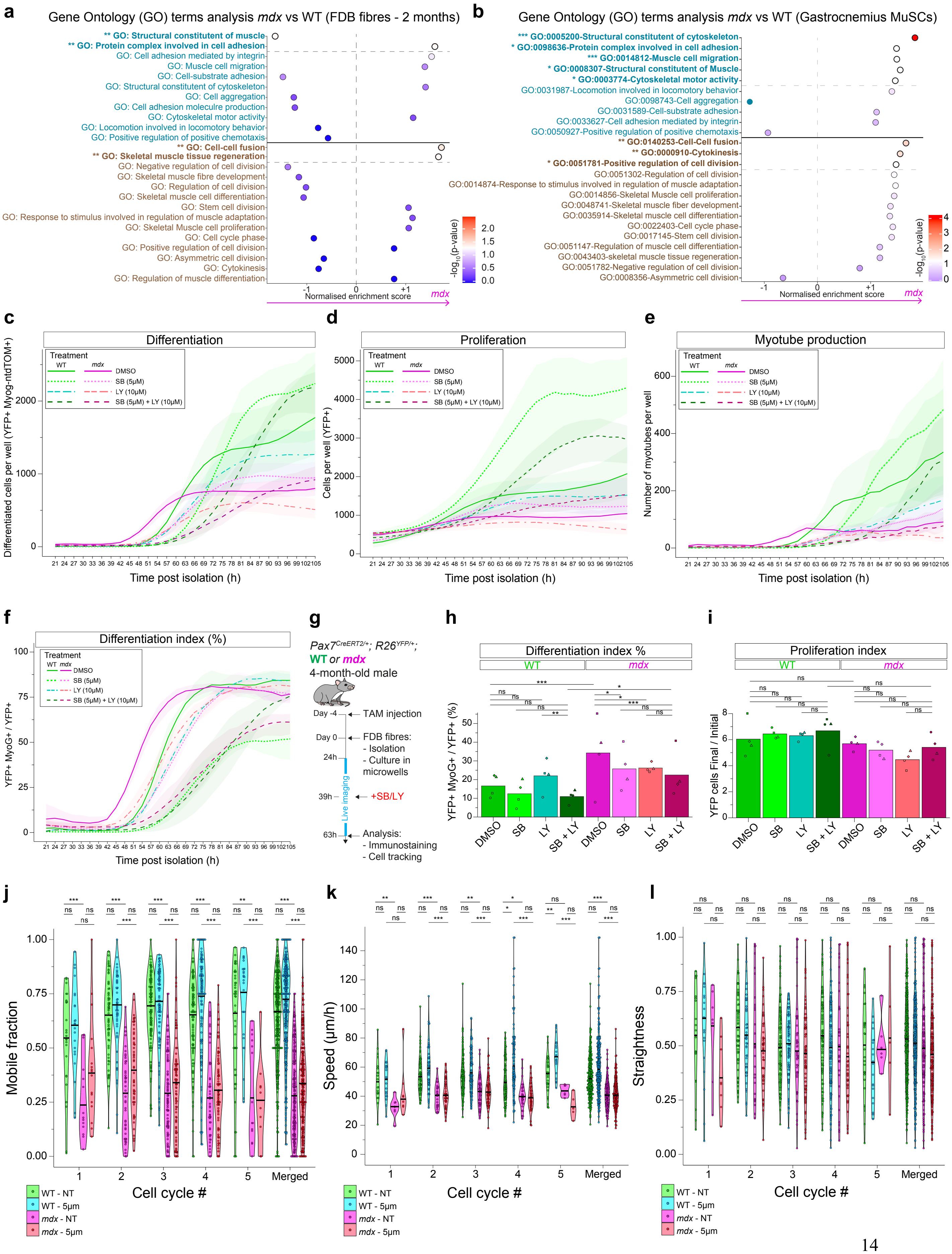
a
Gene Ontology (GO) terms analysis mdx vs WT (FDB fibres - 2 months)
b
Gene Ontology (GO) terms analysis mdx vs WT (Gastrocnemius MuSCs)
Normalised enrichment score
mdx
-log10(p-value)
c
Differentiation
Differentiated cells per well (YFP+ Myog-ntdTOM+)
d
Proliferation
Cells per well (YFP+)
e
Myotube production
Number of myotubes per well
Time post isolation (h)
Treatment
WT mdx
DMSO
SB (5μM)
LY (10μM)
SB (5μM) + LY (10μM)
f
Differentiation index (%)
YFP+ MyoG+ / YFP+
g
Pax7CreERT2/+; R26YFP/+;
WT or mdx
4-month-old male
Day -4 TAM injection
Day 0 FDB fibres: - Isolation - Culture in microwells
24h
39h +SB/LY
Live imaging
63h Analysis: - Immunostaining - Cell tracking
h
Differentiation index %
YFP+ MyoG+ / YFP+ (%)
i
Proliferation index
YFP cells Final / Initial
WT
mdx
DMSO
SB
LY
SB + LY
j
Mobile fraction
k
Speed (μm/h)
l
Straightness
Cell cycle #
Merged
WT - NT
WT - 5μm
mdx - NT
mdx - 5μm

**Supplementary Figure 6.** Related to Figure 4.

**(a)** Gene Ontology (GO) enrichment analysis of FDB fibres RNA-seq data from 2-month-old WT and *mdx* mice[43], focused on terms related to migration and proliferation/differentiation.

**(b)** GO enrichment analysis of gastrocnemius muscle scRNA-seq data from 2-month-old WT and *mdx* mice[7], focused on terms related to migration and proliferation/differentiation in MuSCs.

**(c)** Kinetics of differentiated cell production of WT and *mdx* cells with SB/LY inhibitors. N = 3 experiments.

**(d)** Kinetics of total cell production of WT and *mdx* cells with SB/LY inhibitors. N = 3 experiments.

**(e)** Kinetics of myotube production of WT and *mdx* cells with SB/LY inhibitors. N = 3 experiments.

**(f)** Kinetics of differentiation index of WT and *mdx* cells with SB/LY inhibitors. N = 3 experiments.

**(g)** Experimental scheme. Also see Methods.

**(h)** Differentiation index of WT and *mdx* MuSCs (~63h of culture) with SB and/or LY. p (WT_Ctr vs *mdx*_Ctr) = 1.56e-07; p (WT_Ctr vs WT_SB) = 0.68 ; p (WT_Ctr vs WT_LY) = 0.30; p (WT_Ctr vs WT_SB+LY) = 0.30; p (*mdx*_Ctr vs *mdx*_SB) = 0.022; p (*mdx*_Ctr vs *mdx*_LY) = 0.039; p (*mdx*_Ctr vs *mdx*_SB+LY) = 6.2e-04. N = 4 experiments.

**(i)** Proliferation index of WT and *mdx* MuSCs (~63h of culture) with SB and/or LY. p (WT_Ctr vs *mdx*_Ctr) = 0.46; p (WT_Ctr vs WT_SB) = 0.99; p (WT_Ctr vs WT_LY) = 0.99; p (WT_Ctr vs WT_SB+LY) = 0.89; p (*mdx*_Ctr vs *mdx*_SB) = 0.78; p (*mdx*_Ctr vs *mdx*_LY) = 0.28; p (*mdx*_Ctr vs *mdx*_SB+LY) = 0.95. N = 4 experiments.

**(j)** Mobile fraction of WT and *mdx* MuSCs on isolated FDB fibres, with or without SB, for individual and merged cell cycles. N = 3 experiments.

**(k)** Migration speed of mobile fraction of WT and *mdx* MuSCs on isolated FDB fibres, with or without SB, for individual and merged cell cycles. N = 3 experiments.

**(l)** Migration straightness of mobile fraction of WT and *mdx* MuSCs on isolated FDB fibres, with or without SB, for individual and merged cell cycles. N = 3 experiments.

Statistical tests: **(c-f)** Data are presented as mean values over the 3 replicates (bold) + individual values of each replicates. **(h,i)** Bar plots represent mean values and points represent individual values. **(j-l)** Data are presented as mean values; Horizontal lines in violin plots represent the

mean. **(h-l)** Two-sided test from Linear mixed models with Tukey's correction. * $p < 0.05$, ** $p < 0.01$, *** $p < 0.001$. Source data are provided as a Source Data file.

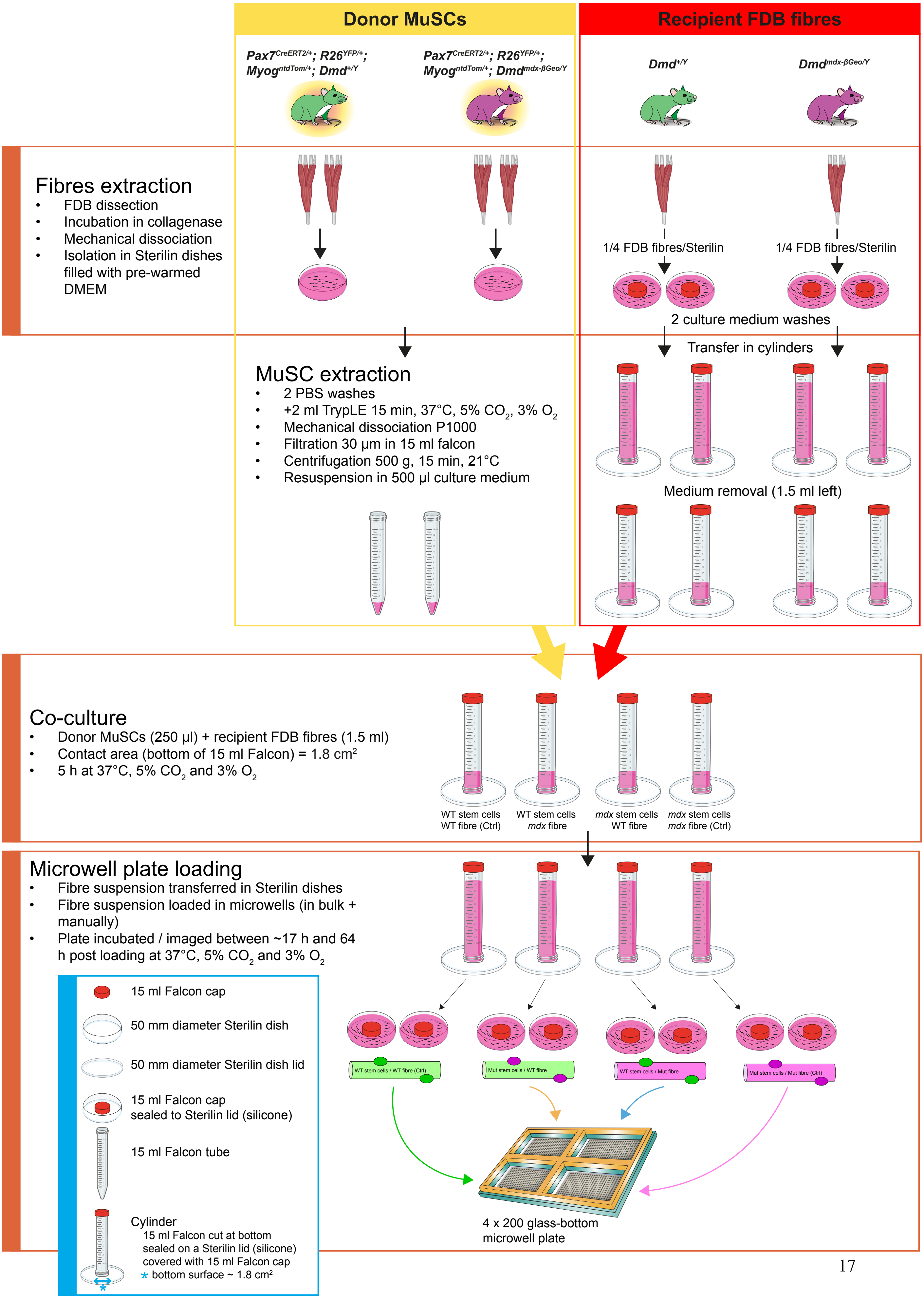
Donor MuSCs
Recipient FDB fibres
*Pax7*$^{CreERT2/+}$; *R26*$^{YFP/+}$; *Myog*$^{ntdTom/+}$; *Dmd*$^{+/Y}$
*Pax7*$^{CreERT2/+}$; *R26*$^{YFP/+}$; *Myog*$^{ntdTom/+}$; *Dmd*$^{mdx\text{-}\beta Geo/Y}$
*Dmd*$^{+/Y}$
*Dmd*$^{mdx\text{-}\beta Geo/Y}$
Fibres extraction
• FDB dissection
• Incubation in collagenase
• Mechanical dissociation
• Isolation in Sterilin dishes filled with pre-warmed DMEM
1/4 FDB fibres/Sterilin
1/4 FDB fibres/Sterilin
2 culture medium washes
Transfer in cylinders
MuSC extraction
• 2 PBS washes
• +2 ml TrypLE 15 min, 37°C, 5% $CO_2$, 3% $O_2$
• Mechanical dissociation P1000
• Filtration 30 µm in 15 ml falcon
• Centrifugation 500 g, 15 min, 21°C
• Resuspension in 500 µl culture medium
Medium removal (1.5 ml left)
Co-culture
• Donor MuSCs (250 µl) + recipient FDB fibres (1.5 ml)
• Contact area (bottom of 15 ml Falcon) = 1.8 $cm^2$
• 5 h at 37°C, 5% $CO_2$ and 3% $O_2$
WT stem cells WT fibre (Ctrl)
WT stem cells *mdx* fibre
*mdx* stem cells WT fibre
*mdx* stem cells *mdx* fibre (Ctrl)
Microwell plate loading
• Fibre suspension transferred in Sterilin dishes
• Fibre suspension loaded in microwells (in bulk + manually)
• Plate incubated / imaged between ~17 h and 64 h post loading at 37°C, 5% $CO_2$ and 3% $O_2$
15 ml Falcon cap
50 mm diameter Sterilin dish
50 mm diameter Sterilin dish lid
15 ml Falcon cap sealed to Sterilin lid (silicone)
15 ml Falcon tube
Cylinder
15 ml Falcon cut at bottom
sealed on a Sterilin lid (silicone)
covered with 15 ml Falcon cap
* bottom surface ~ 1.8 $cm^2$
WT stem cells / WT fibre (Ctrl)
Mut stem cells / WT fibre
WT stem cells / Mut fibre
Mut stem cells / Mut fibre (Ctrl)
4 x 200 glass-bottom microwell plate

**Supplementary Figure 7. Cross-transplantation of MuSCs and FDB fibres.** Related to Figure 3.

Two *Pax7*$^{CreERT2/+}$*; R26*$^{YFP/+}$*; Myog*$^{ntdTom/+}$*; Dmd*$^{+/Y}$ or *Dmd*$^{mdx\text{-}\beta Geo/Y}$ 4-month-old male mice were used as donors of WT or *mdx* YFP+ MuSCs respectively. Two *Dmd*$^{+/Y}$ or *Dmd*$^{mdx\text{-}\beta Geo/Y}$ 4-month-old male mice were used to generate WT or *mdx* recipient FDB fibres. FDB muscles from all mice were dissected and FDB fibres were isolated (see Methods). FDB fibres from donor mice were further treated (TrypLE, mechanical dissociation, filtration) to isolate MuSCs. Recipient FDB fibres mice were transferred in 1.5 ml of medium in cylinders (from Falcon 15 ml tubes). 4 combinations of donor MuSCs with recipient FDB fibres were co-cultured for 5h at 37°C, 5% $CO_2$, 3% $O_2$. Grafted FDB fibres were isolated in a microwell plate, cultured and live-imaged.

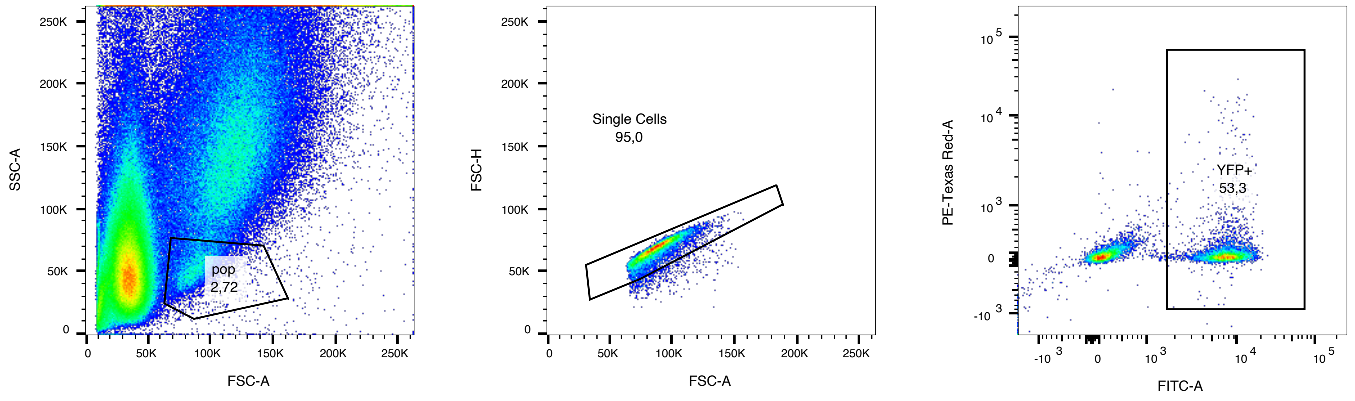


**Supplementary Figure 8. Fluorescence Activated Cell Sorting gating strategy for muscle stem cells.**

MuSCs were analysed (CytoFLEX, Beckman Coulter) or isolated (Aria III, BD Biosciences) based on cell size, granularity (doublets excluded) and YFP fluorescence. Cells from *Pax7*$^{CreERT2/+}$*; R26*$^{YFP/+}$ mice were used to determine the positivity threshold of Myog-ntdTOM.

| Strain | Oligonucleotide | Sequence (5' to 3') | PCR conditions | PCR products |
|---|---|---|---|---|
| *Pax7*$^{CreERT2}$ | Pax7 GaKa mutant rev<br>Pax7 GaKa WT for<br>Pax7 GaKa WT rev | CAAAAGACGGCAATATGGTG<br>GCTGCTGTTGATTACCTGGC<br>CTGCACTGAGACAGGACCG | 95°C 5'<br>(95°C 30''; 55°C 30''; 72°C 1') x40<br>72°C 5' | WT: 419 bp<br>Knock-in: 235 bp |
| *R26*$^{mTmG}$ | CT2 R1 Rosa 1 WT<br>CT2 R3 Rosa 3 WT<br>pCAG R10 | AAAGTCGCTCTGAGTTGTTAT<br>GGAGCGGGAGAAATGGATATG<br>GTCGTTGGGCGGTCAG | 95°C 5'<br>(95°C 30''; 55°C 30''; 72°C 1') x40<br>72°C 5' | WT: 600 bp<br>Knock-in: 350 bp |
| *R26*$^{YFP}$ | CT2 R1 Rosa 1 WT<br>GFP seq2 for<br>Rosa26 F2 | AAAGTCGCTCTGAGTTGTTAT<br>CCGCCCTGAGCAAAGACCCCAACG<br>CAGGTTAGCCTTTAAGCCTGC | 95°C 5'<br>(95°C 30''; 55°C 30''; 72°C 1') x40<br>72°C 5' | WT: 247 bp<br>Knock-in: 392 bp |
| *Myog*$^{ntdTomato}$ | MyoG-nTdT Fwd<br>MyoG-nTdT Rev<br>MyoG-nTdT WT | TTCCTGTACGGCATGGACGAG<br>CAGGACAGCCCCACTTAAAAGC<br>CTTGCTGACCTGAGGGCC | 94°C 2'<br>(94°C 30''; 60°C 30''; 72°C 2') x34<br>72°C 10' | WT: 600 bp<br>Knock-in: 236 bp |
| *Dmd*$^{mdx\text{-}\beta Geo}$ | MDX intron 63R<br>MDX intron 63L<br>MDX 64<br>K03 | GCACGAGCATATGGTTGACACC<br>TAAGTTGAAAAGGTGAGGGC<br>CTCGCGGTTGAGGACAAACTCTTCGC<br>CGCATCGTAACCGTGCATCTGCCAGTTTGA | 95°C 5'<br>(95°C 30''; 55°C 30''; 72°C 1') x40<br>72°C 5' | WT: 200 bp<br>Knock-out: 350 bp |

## Supplementary Table 1. PCR primers

| Reagent or Resource | Concentration | Supplier | Reference |
|---|---|---|---|
| **Antibodies** | | | |
| **Primary antibodies (Immunocytochemistry, ICC)** | | | |
| Mouse monoclonal anti-PAX7 | 1:20 | DSHB | Cat# PAX7 |
| Chicken polyclonal anti-GFP | 1:1000 | Abcam | Cat# ab13970 |
| Mouse monoclonal anti-MYOGENIN | 1:200 | DSHB | Cat# F5D |
| Rabbit polyclonal anti-LAMININ | 1:200 | Sigma | Cat# L9393 |
| **Secondary Antibodies (ICC)** | | | |
| Alexa Fluor 555 F(ab') Goat-anti-Mouse IgG1 | 1:500 | ThermoFisher | Cat# **A-21127** |
| Alexa Fluor 488 F(ab') Goat-anti-Chicken | 1:500 | ThermoFisher | Cat# **A-11039** |
| Alexa Fluor 633 Goat-anti-Rabbit | 1:500 | ThermoFisher | Cat# **A-21070** |
| **Chemicals, Peptides, and Recombinant Proteins** | | | |
| Imalgene 1000® | N/A | XX | XX |
| Rompun 2%® | N/A | XX | XX |
| Cardiotoxin | N/A | Latoxan | Cat# L8102 |
| Tamoxifen | N/A | Sigma | Cat# T5648 |
| Silicone | N/A | Smooth-on | Cat# MoldStar 20T |
| Ethanol 70% | N/A | Sigma | Cat# 32221 |
| Collagenase type 1 | N/A | Sigma | Cat# C0130 |
| Collagenase type 2 | N/A | Serlabo | Car# WOLS04177 |
| DNase I | N/A | Roche | Cat# 11284932001 |
| Ham's F10 | N/A | Sigma | Cat# N6635-10X1L |
| Dispase | N/A | Gibco | Cat# 17105-041 |
| DMEM GlutaMAX | N/A | ThermoFisher | Cat# 31966 |
| Horse Serum | N/A | ThermoFisher | Cat# 11510516 |
| Penicillin/Streptomycin | N/A | ThermoFisher | Cat# 15140122 |
| Chicken Embryo Extract | N/A | Life Science Production | Cat# MD-004D-UK |
| F12 | N/A | Fisher | Cat# 31765027 |
| Fetal MGI: 7442679 Serum | N/A | Fisher | Cat# 10-437-028 |
| Recombinant murine FGF-basic | N/A | PeproTech | Cat# 450-33 |
| Paraformaldehyde | N/A | Euromedex | Cat# 15710 |
| Hepes | N/A | Sigma | Cat# 51558-50ML |
| Goat Serum | N/A | ThermoFisher | Cat# 11540526 |
| Hoechst 33342 | N/A | ThermoFisher | Cat# H1399 |
| Triton | N/A | Sigma | Cat# T8787-250ml |
| O.C.T | N/A | Sakura Finetek | Cat# 4583 |
| Isopentane | N/A | Dutscher | Cat# 24872.298 |
| Bovine Serum Albumin | N/A | Dutscher | Cat# P06-1391100 |
| TrypLE Express Enzyme | N/A | ThermoFisher | Cat# 10718463 |
| p38 MAPK inhibitor - SB 203580 | N/A | Sigma | Cat# 559389-5MG |
| PI3K inhibitors - LY 294002 | N/A | Cell signaling | Cat# 9901S |
| **Experimental Models: Organisms/Strains** | | | |
| Mouse *R26*$^{mTmG}$ | N/A | (Muzumdar et al., 2007) | MGI:3716464 |
| Mouse *Pax7*$^{CreERT2}$ | N/A | (Murphy et al., 2011) | MGI:5141477 |
| Mouse Rosa$^{YFP}$ | N/A | (Srinivas et al., 2001) | MGI: J:80963 |
| Mouse Dmd$^{mdx\text{-}\beta Geo}$ | N/A | (Wertz & Füchtbauer, 1998) | MGI: 94909 |
| Mouse Myogenin$^{ntdTomato}$ | N/A | (Benavente-Diaz et al., 2021) | MGI:7442679 |
| Mouse B6D2F1J | N/A | Janvier Labs | Cat# B6D2F1/JRj |
| **Software and Algorithms** | | | |
| Trackmate | N/A | (Tinevez et al., 2017) | N/A |
| Imaris 7.2.1 | N/A | Bitplane | N/A |
| ImageJ | N/A | N/A | N/A |

**Supplementary Table 2. Reagents**
