# Supplementary material for: Impaired stem cell migration and divisions in Duchenne muscular dystrophy revealed by live imaging
Source: Nat Commun. 2026 Jan 28;17:1769. doi: 10.1038/s41467-026-68474-5 (PMC12917047; doi:10.1038/s41467-026-68474-5)
Supplement: Supplementary file 2 — Description of Additional Supplementary Files [file 41467_2026_68474_MOESM2_ESM.pdf]

Description of Additional Supplementary Files

File Name: Supplementary Movie 1
Description: Intravital imaging of uninjured FDBs at 3 dpi of *Pax7*$^{CreERT2/+}$*; R26*$^{mTmG/+}$*; Dmd*$^{+/Y}$ (WT, top) and *Dmd*$^{mdx\text{-}\beta Geo/Y}$ (*mdx,* bottom*)* adult mice. Related to Figure 1.

File Name: Supplementary Movie 2
Description: Intravital imaging of uninjured FDBs at 3 dpi of *Pax7*$^{CreERT2/+}$*; R26*$^{mTmG/+}$*; Dmd*$^{+/Y}$ (WT, top) and *Dmd*$^{mdx\text{-}\beta Geo/Y}$ (*mdx,* bottom*)* adult mice. High magnification of Movie S1 showing static and migrating myoblasts. Related to Figure 1.

File Name: Supplementary Movie 3
Description: Intravital imaging of injured FDBs at 3 dpi of *Pax7*$^{CreERT2/+}$*; R26*$^{mTmG/+}$*; Dmd*$^{+/Y}$ (WT, top) and *Dmd*$^{mdx\text{-}\beta Geo/Y}$ (*mdx,* bottom*)* adult mice. Related to Figure 1.

File Name: Supplementary Movie 4
Description: Intravital imaging of injured FDBs at 3 dpi of *Pax7*$^{CreERT2/+}$*; R26*$^{mTmG/+}$*; Dmd*$^{+/Y}$ (WT, left) and *Dmd*$^{mdx\text{-}\beta Geo/Y}$ (*mdx*, right*)* adult mice. High magnification of Movie S3 showing migrating myoblasts. Related to Figure 1.

File Name: Supplementary Movie 5
Description: Tracking data overlay of intravital imaging of injured FDBs at 3 dpi of *Pax7*$^{CreERT2/+}$*; R26*$^{mTmG/+}$*; Dmd*$^{+/Y}$ (WT, top) and *Dmd*$^{mdx\text{-}\beta Geo/Y}$ (*mdx,* bottom*)* adult mice. Related to Movie S3.

File Name: Supplementary Movie 6
Description: Tracking data overlay of intravital imaging of injured FDBs at 3 dpi of *Pax7*$^{CreERT2/+}$*; R26*$^{mTmG/+}$*; Dmd*$^{+/Y}$ (WT, top) and *Dmd*$^{mdx\text{-}\beta Geo/Y}$ (*mdx,* bottom*)* adult mice. Related to Movie S4.

File Name: Supplementary Movie 7
Description: Live imaging of FDB fibres and associated MuSCs in microwells from an adult *Pax7*$^{CreERT2/+}$*; R26*$^{mTmG/+}$ mouse. Related to Figure 2.

File Name: Supplementary Movie 8
Description: Representative migration of mobile (top) and static (bottom) YFP-labelled myogenic cells on FDB fibres from a *Pax7*$^{CreERT2/+}$*; R26*$^{YFP/+}$*; Myog*$^{ntdTom/+}$*; Dmd*$^{+/Y}$ mouse. Related to Figure 2.

File Name: Supplementary Movie 9
Description: Representative example of live imaging of a WT FDB fibre grafted with an *mdx* YFP; Myog-ntdTOM-labelled MuSC. Related to Figure 3.

File Name: Supplementary Movie 10
Description: Live-imaging of MuSCs from hindlimbs of *Pax7*$^{CreERT2/+}$*; R26*$^{YFP/+}$*; Myog*$^{ntdTom/+}$*; Dmd*$^{+/Y}$ (WT, left) and *Dmd*$^{mdx\text{-}\beta Geo/Y}$ (*mdx,* right*)* mice isolated and cultured in 96 well plate. Related to Figure 4.
