# Supplementary material for: Impaired stem cell migration and divisions in Duchenne muscular dystrophy revealed by live imaging
Source: Nat Commun. 2026 Jan 28;17:1769. doi: 10.1038/s41467-026-68474-5 (PMC12917047; doi:10.1038/s41467-026-68474-5)
Supplement: Supplementary file 13 — Reporting Summary [file 41467_2026_68474_MOESM13_ESM.pdf]

nature portfolio

Corresponding author(s): Brendan EVANO, Shahragim TAJBAKHSH

Last updated by author(s): Dec 12, 2025

Reporting Summary

Nature Portfolio wishes to improve the reproducibility of the work that we publish. This form provides structure for consistency and transparency in reporting. For further information on Nature Portfolio policies, see our Editorial Policies and the Editorial Policy Checklist.

Statistics

For all statistical analyses, confirm that the following items are present in the figure legend, table legend, main text, or Methods section.

n/a	Confirmed	
☐	☒	The exact sample size (*n*) for each experimental group/condition, given as a discrete number and unit of measurement
☐	☒	A statement on whether measurements were taken from distinct samples or whether the same sample was measured repeatedly
☐	☒	The statistical test(s) used AND whether they are one- or two-sided<br>*Only common tests should be described solely by name; describe more complex techniques in the Methods section.*
☐	☒	A description of all covariates tested
☐	☒	A description of any assumptions or corrections, such as tests of normality and adjustment for multiple comparisons
☐	☒	A full description of the statistical parameters including central tendency (e.g. means) or other basic estimates (e.g. regression coefficient) AND variation (e.g. standard deviation) or associated estimates of uncertainty (e.g. confidence intervals)
☐	☒	For null hypothesis testing, the test statistic (e.g. *F*, *t*, *r*) with confidence intervals, effect sizes, degrees of freedom and *P* value noted<br>*Give P values as exact values whenever suitable.*
☒	☐	For Bayesian analysis, information on the choice of priors and Markov chain Monte Carlo settings
☐	☒	For hierarchical and complex designs, identification of the appropriate level for tests and full reporting of outcomes
☒	☐	Estimates of effect sizes (e.g. Cohen's *d*, Pearson's *r*), indicating how they were calculated

Our web collection on statistics for biologists contains articles on many of the points above.

Software and code

Policy information about availability of computer code

Data collection	Zen (Zeiss) was used to collect widefield imaging data. ImSpector was used to collect intravital imaging data.
Data analysis	The codes used for data analysis are available in the following open source repository:<br>https://gitlab.pasteur.fr/hub/sarde_et_al_2025 and https://doi.org/10.5281/zenodo.17855838

For manuscripts utilizing custom algorithms or software that are central to the research but not yet described in published literature, software must be made available to editors and reviewers. We strongly encourage code deposition in a community repository (e.g. GitHub). See the Nature Portfolio guidelines for submitting code & software for further information.

Data

Policy information about availability of data

All manuscripts must include a data availability statement. This statement should provide the following information, where applicable:

- Accession codes, unique identifiers, or web links for publicly available datasets
- A description of any restrictions on data availability
- For clinical datasets or third party data, please ensure that the statement adheres to our policy

All analysis workflows, representative movies, and tracking datasets are publicly accessible through the GitLab repository cited above. Raw imaging data will be shared upon reasonable request via institutional transfer (e.g., Globus/Nextcloud) along with a detailed data dictionary for reproducibility and benchmarking.

Research involving human participants, their data, or biological material

Policy information about studies with human participants or human data. See also policy information about sex, gender (identity/presentation), and sexual orientation and race, ethnicity and racism.

Reporting on sex and gender	n/a
Reporting on race, ethnicity, or other socially relevant groupings	n/a
Population characteristics	n/a
Recruitment	n/a
Ethics oversight	n/a

Note that full information on the approval of the study protocol must also be provided in the manuscript.

Field-specific reporting

Please select the one below that is the best fit for your research. If you are not sure, read the appropriate sections before making your selection.

[x] Life sciences [ ] Behavioural & social sciences [ ] Ecological, evolutionary & environmental sciences

For a reference copy of the document with all sections, see nature.com/documents/nr-reporting-summary-flat.pdf

Life sciences study design

All studies must disclose on these points even when the disclosure is negative.

Sample size	No formal sample size calculation was conducted. Sample sizes were determined according to the ex vivo and in vivo imaging capacities, ensuring that each imaging session provided sufficient data to support robust statistical analyses. Appropriate statistical models were employed.
Data exclusions	No data were excluded.
Replication	All attempts at replication were successful, in particular cell tracking which was cross-validated between two authors (LS, BE).
Randomization	Each experimental group contained 1 mutant and 1 WT male mice from the same litter and cage to reduce confounding factors.
Blinding	Blinding was not possible because samples (tissue, muscle fibres) have a well recognisable aspect in mutant mice. However, cell tracking was cross checked and verified randomlyfrom WT and mutant samples.

Reporting for specific materials, systems and methods

We require information from authors about some types of materials, experimental systems and methods used in many studies. Here, indicate whether each material, system or method listed is relevant to your study. If you are not sure if a list item applies to your research, read the appropriate section before selecting a response.

Materials & experimental systems

n/a	Involved in the study	
[ ]	[x]	Antibodies
[x]	[ ]	Eukaryotic cell lines
[x]	[ ]	Palaeontology and archaeology
[ ]	[x]	Animals and other organisms
[x]	[ ]	Clinical data
[x]	[ ]	Dual use research of concern
[x]	[ ]	Plants

Methods

n/a	Involved in the study	
[x]	[ ]	ChIP-seq
[ ]	[x]	Flow cytometry
[x]	[ ]	MRI-based neuroimaging

Antibodies

Antibodies used	Mouse monoclonal anti-PAX7, DSHB, Cat# PAX7<br>Chicken polyclonal anti-GFP, Abcam, Cat# ab13970<br>Mouse monoclonal anti-MYOGENIN, DSHB, Cat# F5D<br>Rabbit polyclonal anti-LAMININ, Sigma, Cat# L9393

	Alexa Fluor 555 F(ab') Goat-anti-Mouse IgG1, ThermoFisher, Cat# A-21127<br>Alexa Fluor 488 F(ab') Goat-anti-Chicken, ThermoFisher, Cat# A-11039<br>Alexa Fluor 633 Goat-anti-Rabbit, ThermoFisher, Cat# A-21070
Validation	Primary antibodies for Pax7 and Myogenin have been validated on reporter myoblasts (Pax7-nGFP, Myog-tdTomato). Primary antibody against GFP has been validated with GFP- and GFP+ myoblasts. Laminin antibody has been validated on muscle sections with expected staining pattern (delineating muscle fibres).

Animals and other research organisms

Policy information about studies involving animals; ARRIVE guidelines recommended for reporting animal research, and Sex and Gender in Research

Laboratory animals	Mus musculus. Strains Pax7CreERT2, R26mTmG, R26YFP, MyogntdTom and Dmdmdx-βGeo. 4-month-old males were used.
Wild animals	The study did not involve wild animals
Reporting on sex	Study was investigated in males only as Duchenne Muscular Dystrophy is an X-linked disease affecting males only.
Field-collected samples	The study did not involve samples collected from the field.
Ethics oversight	Animals were handled according to national and European community guidelines, and protocols were approved by the ethics committee (CETEA, comité d'éthique en expérimentation animale) at Institut Pasteur and the french Ministry of Higher Education and Research (Licence 2015-0008 and DAP 220077).

Note that full information on the approval of the study protocol must also be provided in the manuscript.

Plants

Seed stocks	n/a
Novel plant genotypes	n/a
Authentication	n/a

Flow Cytometry

Plots

Confirm that:

- [x] The axis labels state the marker and fluorochrome used (e.g. CD4-FITC).
- [x] The axis scales are clearly visible. Include numbers along axes only for bottom left plot of group (a 'group' is an analysis of identical markers).
- [x] All plots are contour plots with outliers or pseudocolor plots.
- [x] A numerical value for number of cells or percentage (with statistics) is provided.

Methodology

Sample preparation	FDB or hindlimb muscles were dissected and minced in a drop of cold collagenase type 2 (1000U/ml, WOLS04177, Serlabo) in Muscle Dissociation Buffer (MuDB, Nutrient mixture Ham F10 (N6635, Sigma), 10% heat-inactivated Horse serum (HS, 11510516, ThermoFisher), supplemented with NaHCO3 to pH7.4 in distilled water). Samples were incubated in 10 ml of a collagenase 2/MuDB for 90 min at 37°C in a water bath under gentle agitation (70 rpm). Samples were mechanically dissociated (25 ml pipette), MuDB was added up to 50 ml and samples were spun (10 min, 500 g at RT). The supernatant was removed up to 20 ml, cell pellet was resuspended and 1 ml of Dispase (1.8 U/ml in MuDB, 17105-041, Gibco) and 20 µl DNase I (10 mg/ml in DMEM, 11284932001, Roche) were added. Samples were incubated for 90 min at 37°C in a water bath under gentle agitation (70 rpm). Samples were passed through a syringe (Agani Needle 20G, 050109B Fisher Scientific) 10 times, filtered through 40 µm strainer (352235, Dutscher), spun (10 min, 500 g at 4°C) and resuspended in 500 µl of DMEM/2% HS before cytometry.
Instrument	Analyses were performed on a CytoFLEX (Beckman Coulter) and sorting on an Aria III (BD Biosciences).
Software	CytExpert was used on CytoFLEX, Diva on Aria III. FACS data was analysed with FlowJo

April 2023

Cell population abundance	Post-sort purity is routinely verified (second FACS analysis or Pax7 staining) and is >90%.
Gating strategy	MuSCs were isolated/analysed based on cell size and granularity (with doublet exclusion) and YFP fluorescence

☒ Tick this box to confirm that a figure exemplifying the gating strategy is provided in the Supplementary Information.
